# Preoperative indication for systemic therapy extended to patients with early-stage breast cancer using multiparametric 7-tesla breast MRI

**DOI:** 10.1371/journal.pone.0183855

**Published:** 2017-09-26

**Authors:** A. M. T. Schmitz, W. B. Veldhuis, M. B. E. Menke-Pluijmers, W. J. M. van der Kemp, T. A. van der Velden, M. A. Viergever, W. P. T. M. Mali, M. C. J. M. Kock, P. J. Westenend, D. W. J. Klomp, K. G. A. Gilhuijs

**Affiliations:** 1 Department of Radiology / Image Sciences Institute, University Medical Center Utrecht, Utrecht, the Netherlands; 2 Department of Surgery, Albert Schweitzer Hospital, Dordrecht, the Netherlands; 3 Department of Radiology, Albert Schweitzer Hospital, Dordrecht, the Netherlands; 4 Department of Pathology, Albert Schweitzer Hospital, Dordrecht, the Netherlands; University of South Alabama Mitchell Cancer Institute, UNITED STATES

## Abstract

**Purpose:**

To establish a preoperative decision model for accurate indication of systemic therapy in early-stage breast cancer using multiparametric MRI at 7-tesla field strength.

**Materials and methods:**

Patients eligible for breast-conserving therapy were consecutively included. Patients underwent conventional diagnostic workup and one preoperative multiparametric 7-tesla breast MRI. The postoperative (gold standard) indication for systemic therapy was established from resected tumor and lymph-node tissue, based on 10-year risk-estimates of breast cancer mortality and relapse using Adjuvant! Online. Preoperative indication was estimated using similar guidelines, but from conventional diagnostic workup. Agreement was established between preoperative and postoperative indication, and MRI-characteristics used to improve agreement. MRI-characteristics included phospomonoester/phosphodiester (PME/PDE) ratio on 31-phosphorus spectroscopy (^31^P-MRS), apparent diffusion coefficients on diffusion-weighted imaging, and tumor size on dynamic contrast-enhanced (DCE)-MRI. A decision model was built to estimate the postoperative indication from preoperatively available data.

**Results:**

We included 46 women (age: 43-74yrs) with 48 invasive carcinomas. Postoperatively, 20 patients (43%) had positive, and 26 patients (57%) negative indication for systemic therapy. Using conventional workup, positive preoperative indication agreed excellently with positive postoperative indication (N = 8/8; 100%). Negative preoperative indication was correct in only 26/38 (68%) patients. However, ^31^P-MRS score (p = 0.030) and tumor size (p = 0.002) were associated with the postoperative indication. The decision model shows that negative indication is correct in 21/22 (96%) patients when exempting tumors larger than 2.0cm on DCE-MRI or with PME>PDE ratios at ^31^P-MRS.

**Conclusions:**

Preoperatively, positive indication for systemic therapy is highly accurate. Negative indication is highly accurate (96%) for tumors sized ≤2,0cm on DCE-MRI and with PME≤PDE ratios on ^31^P-MRS.

## Introduction

In the past decades, breast cancer treatment has become less invasive, for example from mastectomy to breast conserving therapy, without compromising overall disease-free survival [1,2]. Further progress in individualized treatment involves changing the order of treatment, for example preoperative radiotherapy [3] or preoperative chemotherapy [4], yielding the advantage to monitor tumor response in vivo. Also, more experimental minimally invasive tumor ablation techniques are currently investigated, such as radio-frequency ablation[5], cryoablation[6], and high-intensity focused ultrasound[7,8]. Progress is, however, hampered because the postoperative resection specimen—the golden standard for tumor characterization—is no longer available to guide treatment. Preoperative tumor biopsy, combined with conventional breast imaging, and with assessment of lymph nodes with ultrasound and fine-needle aspiration, shows discordance with postoperative assessment of the resection specimen. This discordance is as high as 40% for tumor grade and mitotic count[9,10], and impacts the ability to accurately omit systemic therapy. Consequently, current guidelines for the indication of systemic therapy cannot easily be translated to preoperative setting for early-stage breast cancer[11]. As a result, early-stage breast cancer patients do not benefit from the advantages of neoadjuvant treatments tailored to the response of cancer. Hence, more accurate preoperative characterization is desirable to reach the same level of confidence as that from a resection specimen.

Magnetic resonance imaging (MRI) visualizes several aspects of tumor biology. For instance, dynamic contrast-enhanced (DCE)-MRI indirectly visualizes angiogenesis[12], and is typically used for local tumor staging. MR diffusion-weighted imaging (MR-DWI) visualizes the ability of water molecules to move freely inside tumors, providing a measure of how chaotic cells have been laid out. It has been investigated for several applications[13,14], including cancer differentiation, although results were not consistent[15–18]. Thirdly, magnetic resonance spectroscopy (MRS) visualizes the metabolism of cancers. In particular, 31-Phosphorus MR spectroscopy (^31^P-MRS) measures the phosphorus components in tumors, which play an important role in the forming of new cell membranes[19]. However, conventional MRI scanners lack the ability to do ^31^P-MRS, because phosphorus components are not highly abundant in the human body. Recent MRI scanners with high magnetic field strength at 7 tesla (T) have been able to detect these components in breast cancers.

In order to increase the confidence of preoperative indication for systemic therapy to the same level as that obtained from the surgically excised tissue, we hypothesized that combining several MRI modalities (i.e., multiparametric imaging) prior to surgery provides complementary information. The objective of this study is to use multiparametric breast MRI at high field strength to preoperatively discriminate between patients with early-stage breast cancer indicated for systemic therapy and patients who are not indicated.

## Materials and methods

Patients were included from the PROFILE (*P*atient *R*isk based *O*n *F*unctional MR*I*) study. All women in this study had histologically proven invasive breast cancer eligible for breast conserving therapy based on conventional imaging. Exclusion criteria were prior surgery, prior radiotherapy of the ipsilateral breast, neoadjuvant chemotherapy, and typical contraindications for MRI. Patients were consecutively recruited from the University Medical Center Utrecht and the Albert Schweitzer hospital in Dordrecht. Approval for this study was obtained from the institutional review board of the University Medical Center Utrecht and written informed consent was obtained from all patients. A total of 46 patients are described in this study, of which 14 patients were reported earlier in an explorative study considering the potential of multiparametric 7-tesla breast MRI to characterize breast cancer [18].

### Conventional diagnostic workup

Preoperatively, tumor size was assessed by the largest tumor diameter on ultrasound or mammographic imaging. Ultrasound-guided 14-Gauge tumor biopsies of the invasive lesions were acquired, and histopathology was obtained. Biopsy-derived tumor tissue was stained using hematoxyline and eosine (H&E). Tumor grade was assessed according to the modified Bloom and Richardson guidelines[20]. Mitotic count was assessed as the number of mitotic figures per 2 mm^2^. Tumor type and estrogen receptor (ER) status were assessed on H&E slides. For ER, a 10% staining threshold was used to differentiate between a positive (≥10%) and a negative (<10%) status. Preoperative lymph node status was assessed using ultrasound-guided fine-needle aspiration of suspected lymph nodes (cortex-thickness >2.3 mm).

Postoperatively, the resection specimen was treated according to a protocol adapted from Egan et al. [21]as described earlier[18]. In short, the resection specimen was cut in approximately 4 mm thick slices and fixed in formalin overnight. The slice containing the largest tumor diameter was chosen as representative for tumor characterization. Tumor size was assessed macro and microscopically. Tumor grade, mitotic count, tumor type, and receptor status was examined using procedures comparable with the assessment of the biopsy-derived tissue. A positive lymph node was defined as a lymph node containing macro or microscopic disease on sentinel node biopsy and/or axillary lymph node dissection when available.

### Indication for systemic therapy

The risk of 10-year mortality and 10-year relapse were estimated using the web-based tool Adjuvant Online (AOL) (version 8.0), which is based on the SEER database and validated in multiple countries[22–24]. The AOL estimates were obtained twice for each patient, once using preoperative information only and once using postoperative information. Hence, patient age at diagnosis, and characteristics from the primary tumor were entered from preoperative or postoperative assessment: ER-status, tumor grade, tumor size, and number of positive lymph nodes. Co-morbidity was set to default: ‘minor problems’. Missing data was set to ‘undefined’. The indication for systemic therapy was based on the Dutch national guidelines (www.oncoline.nl). In accordance with these guidelines an indication for systemic therapy was given when either the predicted risk of 10-year mortality was equal or higher than 15%, or when the predicted risk of 10-year relapse was equal or higher than 25%.

### MRI characteristics

Both DWI and DCE-MRI were performed in prone patient orientation using either a bilateral two-channel transmit/receive breast coil (i.e., setup 1) [25]or a bilateral two-channel transmit and 26-channel receive breast coil (i.e., setup 2) (MR Coils BV, Drunen, the Netherlands) [26]. All imaging was performed on a 7T whole body MR System (Philips Healthcare, Cleveland, Ohio, USA).

#### DWI

For DWI, a Multi slice Spin Echo-EPI was obtained: TE 49 ms, FOV 350 x 160 x 50 mm^3^; acquired resolution 2.0 x 2.0 x 3.0 mm^3^; b-values: 0, 100, 200, 500, 1000 s/mm^2^; scan duration 140–170s. A SENSE acceleration of 3 was performed for setup 2. Fat suppression was obtained using fat-selective adiabatic inversion recovery with an inversion delay of 320ms. Apparent diffusion coefficient (ADC) maps were calculated using all acquired b-values. On ADC maps, tumors were only scored when proper anatomic visualization of tumors and/or surrounding parenchyma was confirmed. ADC was manually drawn at the hypo-intense area of tumors using a region of interest (ROI) of 16–25 mm^2^. Artifacts and areas of necrosis were avoided.

#### DCE-MRI

DCE-MR imaging consisted of six fat-suppressed series, one prior to the injection of 0.1 mmol/kg gadolinium-containing contrast agent (Gadobutrol, Bayer Schering Pharma AG, Berlin, Germany), and five series following injection. The protocol consisted of 3D T1-weighted gradient echo sequences for setup 1 (TR/TE 4.3/2.1ms, flip angle 15°, field-of-view (FOV) 350 x 160 x 160 mm^3^, acquired resolution 1.0 x 1.0 x 1.0 mm^3^, scan duration 108s) and for setup 2 (TR/TE 5.8/2.5ms, flip angle 15°, FOV 350 x 160 x 160 mm^3^, acquired resolution 0.7 x 0.7 x 0.7 mm^3^, scan duration 91s, SENSE acceleration 4 x 2). A radiologist experienced with breast MRI considered all tumors separately using a scoring form based on the standardized American College of Radiology Breast Imaging Reporting and Data System (ACR BI-RADS)-MRI lexicon [27]. Tumor size was assessed as the largest tumor extent over three orthogonal directions. For the multivariate model, missing value was denoted as the mean value.

#### ^31^P-MRS

^31^P-MRS was acquired with a double-tuned unilateral quadrature RF coil using the AMESING [28]sequence (3D ^31^P multi-echo MRSI sequence using spherical k-space sampling; TR/dTE: 6000/45ms, adiabatic flip angle 90°, FOV 160 x 160 x 160 mm, nominal spatial resolution 20 x 20 x 20 mm^3^, scan duration 1536s). One free induction decay (FID) and 5 full echoes were acquired within one TR, resulting in an FID at 0ms and echoes at 45, 90, 135, 180 and 225ms respectively. On ^31^P-MR spectra, the phosphocreatine signal of the pectoral muscle was acquired to confirm proper functioning of the coil, and spectra were only analyzed when clearly visible. The ^31^P-MR spectra were assessed of the voxel containing the tumor. Two experienced observers (W.K. and D.K.) scored the spectra individually according to a lexicon which was designed previously and in consensus[18]. This lexicon categorizes proliferative activity of tumors on dominance of either phosphomonoester (PME) phosphodiester (PDE) or peaks into three groups (PME<PDE; PME = PDE; PME>PDE). Observers were blinded for patient information and tumor characteristics from histopathology. The spectra of 29 tumors, which are described here for the first time, were scored separately to assess the inter-observer variability. Finally, consensus was obtained between the two readers. For the multivariate model, missing spectra were denoted as the median score (PME = PDE).

### Statistical analysis

Analyses were performed using SPSS software (version 23.0 for Windows; SPSS; Chicago, Ill). The agreement between preoperative and postoperative tumor characteristics (ER-status, tumor grade, tumor size, and lymph node status) was established using two-sided Pearson’s chi-squared and Fisher’s exact tests. For ^31^P-MRS, agreement in terms of inter-observer variability was established from the scores obtained by the two observers using the Kappa statistic. Associations of ADC and ^31^P-MRS score with the postoperative tumor characteristics (ER-status, tumor grade, tumor size, lymph node status, and mitotic count) was assessed using two-sided Pearson’s chi-squared, Fisher’s exact, and Kruskal Wallis tests. The agreement between preoperative and postoperative indication for systemic therapy was expressed using the Kappa statistic. Whether an association existed between the postoperative indication for systemic therapy and tumor size on DCE-MRI, ADC on DWI, or ^31^P-MRS scoring was assessed using two-sided Pearson’s chi-squared and Mann-Whitney U tests.

For the decision model, a multivariate decision tree was built based on the CHAID growing method to estimate the postoperative indication for systemic therapy using preoperatively available characteristics. The (positive/negative) postoperative indication for systemic therapy was taken as the dependent variable. Independent variables included the preoperative indication for systemic therapy (as a forced first variable) and the imaging characteristics with association (p≤0,05) to the postoperative indication for systemic therapy. Conventional diagnostics were prioritized where possible. Backward covariate selection (p-to-remove = 0.05) was deployed.

## Results

### Conventional diagnostic workup

Forty-six patients were included—mean age 60 years (range 43–74 years)–with 48 histologically proven invasive carcinomas in total. In two patients a second ipsilateral tumor was detected.

Postoperatively, 45 lesions had a positive ER-status, and 3 lesions had a negative ER-status. Tumors were stratified into grade 1 (N = 14), grade 2 (N = 30) and grade 3 (N = 4). The mean size of the 48 invasive lesions was 14 mm (range: 3–36 mm). For 16 tumors lymph node metastases were found in either one (N = 11), two (N = 3), or three (N = 2) nodes. For 32 tumors, no positive lymph nodes were found. The mean mitotic count was 5 per 2 mm^2^ (range: 0–38).

The association between preoperative and postoperative tumor characteristics is shown in Table 1. For ER-status high agreement was seen in 47/48 (98%) tumors (Kappa = 0.846). Less agreement was observed for tumor grade, in 35/48 (73%) tumors (Kappa = 0.627), and tumor size, in 37/48 (77%) tumors (Kappa = 0.604). For lymph node status the lowest agreement was observed in 34/48 (71%) tumors (Kappa = 0.106). Although a positive preoperative lymph node status was highly suggestive for a positive postoperative status, a negative status is only correct in 70% of tumors.

**Table 1:** Association between the preoperative and postoperative tumor characteristics on conventional diagnostic workup. ER = Estrogen receptor.

Preoperative	Postoperative				p-value
**ER-status**	*Positive*	*Negative*			
*Positive (n; %)*	**44 (100%)**	0 (0%)			P<0.001
*Negative (n; %)*	1 (25%)	**3 (75%)**			Kappa = 0.846
**Tumor grade**	*Grade 1*	*Grade 2*	*Grade 3*		
*Grade 1 (n; %)*	**12 (71%)**	5 (29%)	0 (0%)		P<0.001
*Grade 2 (n; %)*	2 (7%)	**23 (88%)**	4 (15%)		Kappa = 0.475
*Grade 3 (n; %)*	0 (0%)	2 (100%)	**0 (0%)**		
**Tumor size**	*0*.*1–1*.*0 cm (n; %)*	*1*.*1–2*.*0 cm (n; %)*	*2*.*1–3*.*0 cm (n; %)*	*3*.*1–5*.*0 cm (n; %)*	
*0*.*1–1*.*0 cm (n; %)*	**14 (78%)**	4 (22%)	0 (0%)	0 (0%)	P<0.001
*1*.*1–2*.*0 cm (n; %)*	2 (8%)	**19 (79%)**	2 (8%)	1 (4%)	Kappa = 0.604
*2*.*1–3*.*0 cm (n; %)*	0 (0%)	1 (17%)	**4 (67%)**	1 (17%)	
**Lymph node status**	*Positive*	*Negative*			
*Positive (n; %)*	**2 (100%)**	0 (0%)			p = 0.106
*Negative (n; %)*	14 (30%)	**32 (70%)**			Kappa = 0.160

### MRI characteristics

On DCE-MRI, a mean tumor size of 18 mm (range: 8–51 mm) was seen. Tumor extent could not be clearly assessed in one patient. On DWI, the ADC was successfully assessed in 40 tumors. In six tumors no proper anatomic visualization of tumor and/or surrounding parenchyma was seen: one tumor was outside the chosen field-of-view, and one tumor was not imaged due to a technical problem. The mean ADC of the tumors was 773x10^−6^mm^2^/s (range: 539–1013 x10^−6^mm^2^/s). The ^31^P-MRS was assessed in 40 tumors. One patient stopped prior to the start of the ^31^P-MRS sequence, in four patients ^31^P-MRS was not performed due to technical difficulties, and in three patients no signal from the pectoral muscle was seen on ^31^P-MR spectra. Inter-observer agreement for scoring ^31^P-MRS was found for 24/29 tumors (83%; kappa: 0.716) (Table 2). Observer 2 underscored five tumors as compared with observer 1. In consensus, ^31^P-MRS scoring of observer 1 was maintained. Overall, this led to a stratification of 10 tumors in the PME<PDE group, 13 tumors in the PME = PDE group, and 17 tumors in the PME>PDE group.

**Table 2:** Inter-observer variability between observer 1 (W.K.) and observer 2 (D.K.) for 31-phosphorus spectroscopy (^31^P-MRS) scoring. Agreement is seen in 24/29 tumors (83%; kappa: 0.716). PME = Phosphomonoesters. PDE = Phosphodiesters.

		^**31**^**P-MRS score observer 2**			
		PME<PDE	PME = PDE	PME>PDE	
^**31**^**P-MRS score observer 1**	PME<PDE	**3**	0	0	Kappa: 0.716
	PME = PDE	0	**12**	0	
	PME>PDE	1	4	**9**	

The association of ADC and ^31^P-MRS score with the postoperative tumor characteristics at pathology are shown in Table 3. In short, a significantly (p = 0.041) lower mean ADC was found in ER-positive tumors compared with ER-negative tumors, although the vast majority of tumors were ER-positive. In conformity with results from the prior explorative study, an inverse trend was again seen between ADC and tumor grade (p = 0.085), and for ^31^P-MRS a significant association was observed with mitotic count (p = 0.014).

**Table 3:** Association between postoperative histopathology and 7T MRI characteristics. A significant association (p<0.05*) was seen between the mean apparent diffusion coefficient (ADC) on diffusion weighted imaging and estrogen receptor (ER)-status, and also between the 31-phosphorus magnetic resonance spectroscopy (^31^P-MRS)-scoring and lymph node status as well as mitotic count. A trend (p<0.10 **) was seen between mean ADC and tumor grade as well as lymph node status. PME = Phosphomonoesters. PDE = Phosphodiesters.

*Postoperative*			ADC mean	p-value	Total		^31^P-MRS		p-value
*tumor characteristics*			x10^−6^mm^2^/s (sd)			PME<PDE	PME = PDE	PME>PDE	
**ER-status**									
	*Positive*	N = 37	762 (sd: 114)	0.041*	N = 37	9	12	16	0.925
	*Negative*	N = 3	911 (sd: 84)		N = 3	1	1	1	
**Tumor grade**									
	*Grade 1*	N = 10	809 (sd:109)	0.085**	N = 12	4	5	3	0.401
	*Grade 2*	N = 27	774 (sd:120)		N = 24	5	8	11	
	*Grade 3*	N = 3	647 (sd:32)		N = 4	1	0	3	
**Tumor size**									
	*0*.*1–1*.*0*	N = 12	761 (sd:128)	0.325	N = 15	6	5	4	0.230
	*1*.*1–2*.*0*	N = 21	798 (sd:119)		N = 19	3	8	8	
	*2*.*1–3*.*0*	N = 5	724 (sd:87)		N = 5	1	0	4	
	*3*.*1–5*.*0*	N = 2	715 (sd:130)		N = 1	0	0	1	
**Lymph node status**									
	*Negative*	N = 25	798 (sd: 116)	0.081**	N = 25	7	11	7	0.044*
	*Positive*	N = 15	732 (sd: 114)		N = 15	3	2	10	
**Mitotic count**	mean (sd)	N = 40		R^2^ = 0.077	N = 40	4.5 (5.9)	3.4 (3.3)	8.5 (8.4)	0.014*

For lymph node status, both DWI and ^31^P-MRS showed association with the postoperative tumor characteristics. ADC yielded a trend (p = 0.081), with lower mean ADC in tumors with positive lymph node status. ^31^P-MRS showed a significant correlation with lymph node status (p = 0.044): the majority of tumors with a positive lymph node status had PME>PDE score.

### Indication for systemic therapy

Postoperatively, 20/46 patients (43%) had positive indication for systemic therapy, and 26/46 patients (57%) negative indication (Table 4). Preoperatively, a positive indication for systemic therapy was in agreement with the postoperative indication in 8/8 patients (100%). A negative preoperative indication agreed with the postoperative indication in 26/38 patients (68%).

**Table 4:** Association between the preoperative and postoperative indication for systemic therapy based on conventional diagnostic workup.

Indication systemic therapy	Postoperative			p-value
**Preoperative**	*Positive*	*Negative*	*Missing*	
*Positive (n; %)*	**8 (100%)**	0 (0%)	0	p<0.001
*Negative (n; %)*	12 (32%)	**26 (68%)**	1	Kappa = 0.430

### Indication for systemic therapy and MRI characteristics

Significant association (p = 0.002) was found between the postoperative indication for systemic therapy and tumor size on DCE-MRI (Table 5). Smaller tumors (≤2 cm) were more often associated with a negative indication for systemic therapy, whereas larger tumors (>2 cm) were more often associated with a positive indication. In addition, a significant association (p = 0.030) was observed between the postoperative indication and the ^31^P-MRS score. Tumors with a ^31^P-MRS score PME≤PDE were more often associated with a negative indication, and tumors with PME>PDE more often with a positive indication.

**Table 5:** Univariate association of 7 tesla magnetic resonance imaging (7T MRI) characteristics and postoperative indication for systemic therapy. A significant (p<0.05*) association is seen between the postoperative indication for systemic therapy and dynamic contrast-enhanced (DCE)-MRI as well as 31-phosphorus magnetic resonance spectroscopy (^31^P-MRS). No association was seen between the postoperative indication and the apparent diffusions coefficient (ADC) on diffusion-weighted imaging (DWI).

7T MRI characteristics	Postoperative indication systemic therapy		p-value
	No	Yes	
**DCE-MRI tumor size (cm)**			
*0*.*1–1*.*0 (n; %)*	**6 (100%)**	0 (0%)	p = 0.002*
*1*.*1–2*.*0 (n; %)*	**17 (65%)**	9 (35%)	
*2*.*1–3*.*0 (n; %)*	2 (20%)	**8 (80%)**	
*3*.*1–5*.*0 (n; %)*	0 (0%)	**3 (100%)**	
*missing (n)*	1	**0**	
**DWI (mean ADC x10**^**−6**^**mm**^**2**^**/s; sd)**	803 (N = 19; sd:124)	750 (N = 19; sd: 113)	p = 0.138
*missing (n)*	7	1	
**31P-MRS (score)**			
*PME<PDE (n; %)*	**7 (70%)**	3 (30%)	p = 0.014*
*PME = PDE (n; %)*	**9 (75%)**	3 (25%)	
*PME>PDE (n; %)*	4 (25%)	**12 (75%)**	
*missing (n)*	6	2	

### Preoperative decision model

**Fig 1:**
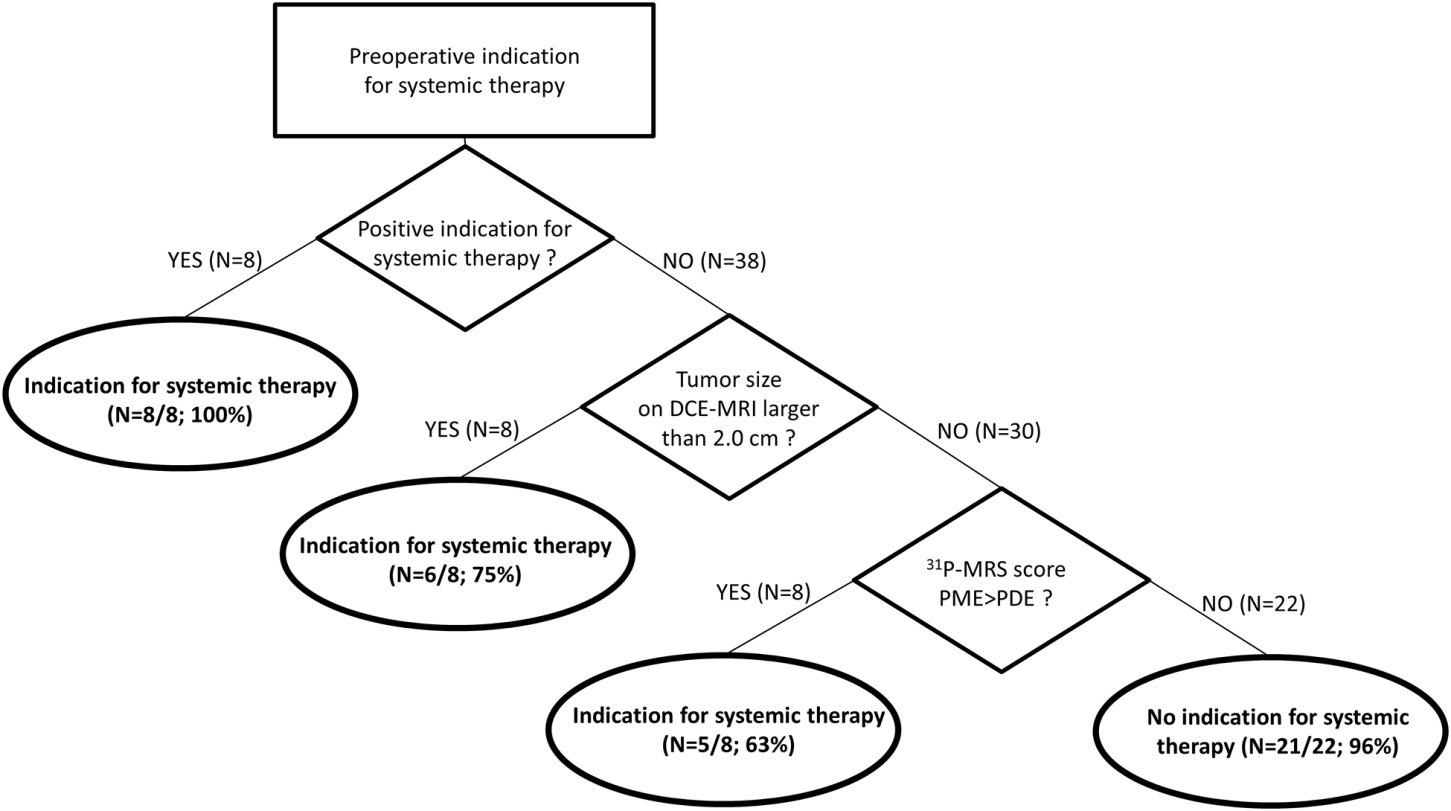
A decision tree was created to establish a preoperative indication for systemic therapy, taking the postoperative indication as the golden standard. 7T DCE-MRI = 7 tesla dynamic contrast-enhanced magnetic resonance imaging. ^31^P -MRS = 31-Phosphorus MR spectroscopy. PME = Phosphomonoesters. PDE = Phosphodiesters.

The decision model to preoperatively assess the indication for systemic therapy is shown in Fig 1. A positive preoperative indication for systemic therapy showed high agreement with a positive postoperative indication (N = 8/8; 100%).

**Fig 2:**
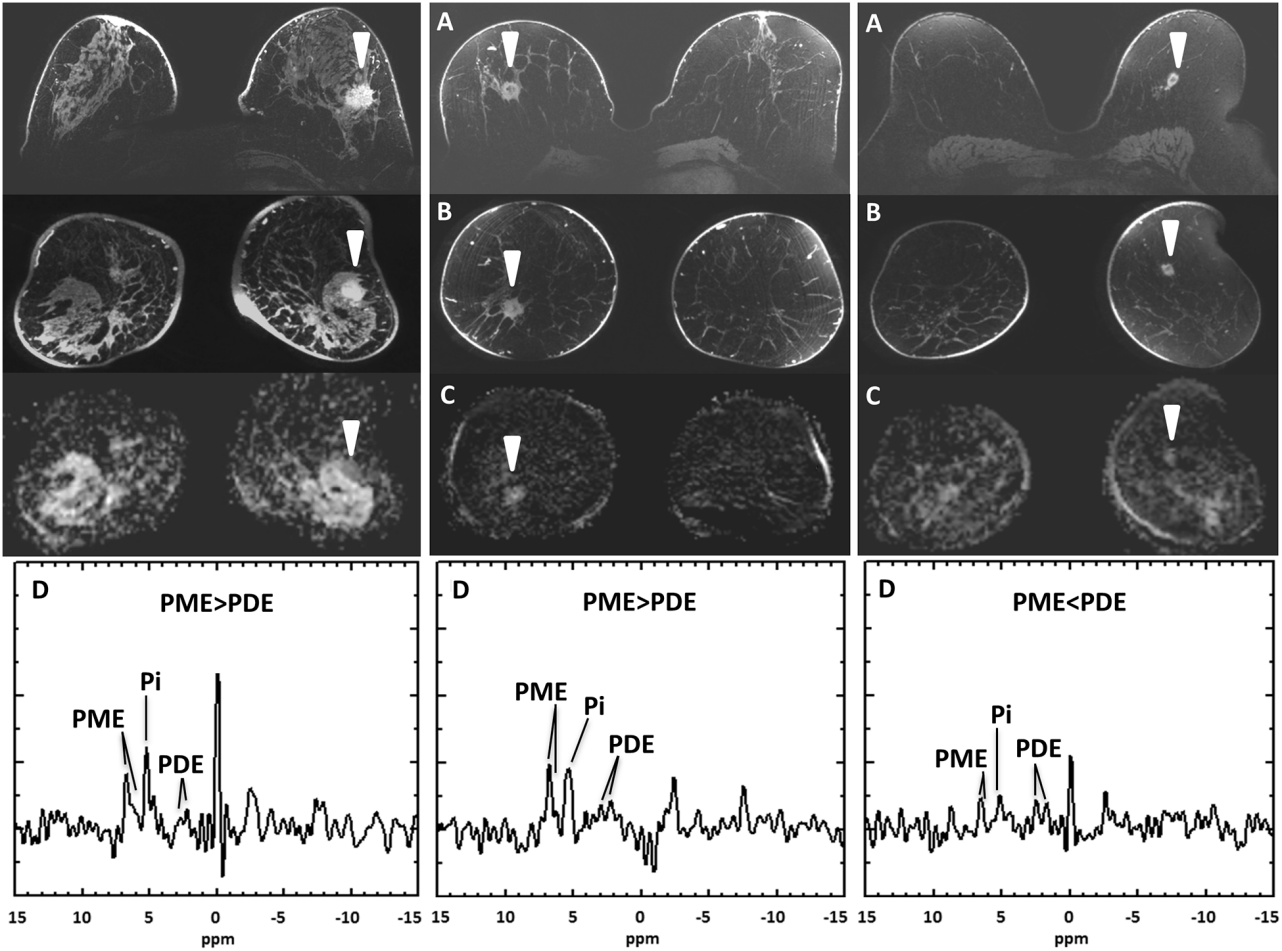
Examples of 7 tesla MR imaging in three patients, showing the first post-contrast dynamic contrast-enhanced MR imaging in the (A) transverse plane and the (B) coronal plane, with (C) the corresponding apparent diffusion coefficient (ADC) map; and (D) the corresponding 31-phosphorus MR spectroscopy (^31^P-MRS) scoring upon the ratio of phosphomonoesters (PME) to phosphodiesters (PDE). All three patients (left/middle/right) had a negative preoperative indication for systemic therapy based on biopsy and conventional breast imaging. On the left, imaging is shown of a 47-year old woman. The decision-tree model upstages this assessment to positive indication: DCE-MRI showed a 23-mm sized lesion, an ADC of 746 x10^−6^mm^2^/s, and a PME>PDE score. Postoperatively, a grade 2 tumor and a positive indication for systemic therapy was established. In the middle, imaging is shown of a 58-year old women. The decision-tree model again upstages this assessment to positive indication: DCE-MRI showed an 18-mm sized lesion, an ADC of 980 x10^−6^mm^2^/s. The MRS score was, however, PME>PDE. Postoperatively, a grade 2 tumor and a positive indication for systemic therapy was established. On the right, imaging is shown of a 71-year old woman. The decision-tree model confirms the negative indication: DCE-MRI showed a 16-mm sized lesion, DWI a mean ADC of 834 x10^−6^mm^2^/s, and ^31^P-MRS a PME<PDE score. Postoperatively, a grade2 tumor and a negative indication for systemic therapy was established. Pi = Inorganic phosphate.

A negative preoperative indication (N = 38) was, however, less accurate. For patients with a negative preoperative indication, only 26/38 patients (68%) had a negative postoperative indication. However, in this group, exclusion of patients with a tumor larger than 2.0cm on DCE-MRI, raised the accuracy for a correct negative indication to 24/30 patients (80%). With the addition of ^31^P-MRS scoring, exclusion of patients with a tumor scoring PME>PDE on ^31^P-MRS, raised the accuracy for a correct negative indication to 21/22 patients (96%). Examples are shown in Fig 2.

## Discussion

Positive preoperative indication for systemic therapy based on biopsied tissue and conventional breast imaging showed high agreement with a positive postoperative indication from resected tissue (the gold standard). A negative preoperative indication was only accurate in tumors not larger than 2.0 cm on DCE-MRI or with a PME>PDE score at ^31^P-MRS. Thus, in a subgroup of patients with early-stage breast cancer, preoperative multiparametric breast MRI at 7T enables an accurate risk assessment of breast cancer comparable to conventional assessment from surgically resected tissue.

These findings may ultimately have practice-changing impact on the treatment of patients with early-stage breast cancer, who are currently susceptible to overtreatment [29]. Continuous effort is made towards more individualized and less invasive therapy in this group of patients, but current guidelines for systemic therapy still require a representative resection specimen. Early-stage breast cancer patients in whom the positive indication for systemic therapy is known with high accuracy prior to surgery could be treated with systemic therapy prior to the surgical intervention, thus achieving similar long-term benefit as postoperative systemic therapy, but with the added benefit of allowing response of the tumor and axilla to be monitored [30]. This opens new treatment paradigms where patients with excellent response may be spared axillary surgery and minimalize the extent of breast surgery, which will reduce side effects and improve the quality of life after treatment [31,32]. Another possible scenario is to substitute surgery on a completely responding tumor with accelerated partial breast irradiation directed at the former tumor bed [33]. For early-stage breast cancer patients with a negative preoperative indication for systemic therapy, multiparametric 7T breast MRI could be added to the clinical workflow. Within this subgroup, patients with tumors of 2,0 cm or smaller on DCE-MRI and PME≤PDE score on ^31^P-MRS may be suited for less invasive treatment, and the addition of a 7T MRI scan in this subgroup may give new impulse to ongoing studies on less- and non-invasive techniques for primary breast cancer treatment, such as MR-guided high-intensity focused ultrasound [7,8].

Guidelines for preoperative (i.e., neoadjuvant) chemotherapy are currently focused on patients for whom little doubt exists that they will be indicated for chemotherapy after inspection of the resection specimen. Consequently, new techniques to extend the indication for preoperative chemotherapy towards early breast cancer must ensure that patients who would be selected postoperatively will also be selected preoperatively. The current study demonstrates the potential to identify three groups of patients preoperatively: those with high confidence that systemic therapy is indicated, those with high confidence that it is not, and a third group where the assessment is uncertain.

The indication for systemic therapy was estimated from 10-year risk estimates of mortality and relapse derived from AOL and considering patient age, ER-status, tumor grade, tumor size, and the number of positive lymph nodes. In the decision model, a subgroup of early breast cancer patients is selected with a negative indication based on tumor size at DCE-MRI and ^31^P-MRS score. Although tumor size on DCE-MRI may be anticipated as an imaging biomarker for patient prognosis, the complementary role of ^31^P-MRS as a biomarker for patient prognosis has been demonstrated for the first time. The results may be explained by the correlation between ^31^P-MRS and mitotic count, where higher levels of PME in the ^31^P-MRS phosphorus metabolism are associated with higher mitotic count. This confirms the finding in a recent explorative study with ^31^P-MRS on 7T MRI [18]. In addition, ^31^P-MRS on 7T MRI was found to be significantly associated with lymph node status in the current study.

Without taking preoperative multiparametric imaging into account, the findings of the current study are in agreement with a previous study performed on an independent database of patients from a different hospital [11]. In that study, a positive preoperative indication showed high agreement with a positive postoperative indication in 94% of patients, and negative indication showed only 67% agreement. In this latter group of patients, additional stratification using preoperative sentinel lymph node biopsy was considered a potential clinical step, which led to an anticipated agreement of approximately 89%. In the current study, however, the addition of tumor size on DCE-MRI and ^31^P-MRS scoring resulted in an agreement of 96%. These results suggest that information from multiparametric imaging has potential to complement the missing lymph node status. Moreover, when considering minimally invasive treatment, a proposed stratification that requires additional imaging would be favored over an additional invasive surgical procedure.

Other techniques are currently available to assess the risk of metastases (i.e., indication for systemic therapy), for example molecular assays such as the Mammaprint [34] or Oncotype-DX [35]. These techniques are increasingly used, and found to be useful prognostic indicators. Nonetheless, molecular essays are typically established from the postoperative resection specimen as well. Although it is currently possible to obtain a Mammaprint and Oncotype-DX assessment from biopsied tissue, it is as yet unclear to witch extent the results are affected by the heterogeneity of the tumor. Moreover, the test may take two weeks to complete which could result in delay of surgery when applied in the context such as described in the current study. Conversely, molecular essays are based on different risk models than the one used in current study, and may result in superior risk assessments if a resection specimen is available. An advantage of multiparametric MRI is, however, that results become available directly after imaging, that it provides information across the entire tumor, and that it is relatively inexpensive compared with molecular tests.

Although tumor size on DCE-MRI can be visualized with conventional 1.5T and 3.0T MRI scanners, imaging, ^31^P-MRS is still limited to 7T MRI. Although not widely available at the moment, 7T MRI could be used as a specialized in-vivo biology-imaging device in a select patient group with early breast cancer to broaden the indication for upfront therapy. DCE-MRI is typically used to define tumor extent, most importantly assessment of tumor size. In this study, DCE-MRI was used in accordance with the regular clinical setting. Prior studies have suggested more advanced imaging analyses to provide relationships between DCE-MRI characteristics and prognostic markers of breast cancer [36]. Whether 7 Tesla Breast MRI has a potential role in this subject will be focus of future study. Also, in future study breast cancer subtype should be taken into consideration as they have an important role in guiding treatment decisions. Unfortunately this was currently not possible due to limited patient numbers.

This study has some limitations. Although substantial agreement was found between observers to interpret ^31^P-MRS, thus making it a reproducible tool, continuous efforts must be made to obtain quantitative assessment of ^31^P-MRS. Also, technical issues in performing ^31^P-MRS occurred in seven patients in this study. For DWI, missing values were seen in six tumors caused by a non-proper visualization of tumors or the surrounding parenchyma. DWI was possibly not included in the decision tree because of these issues, and hence DWI cannot yet be excluded as a prognostic indicator. Ongoing technical research in MR coil design, MR pulse sequence design, and MR image analysis may provide solutions to overcome these limitations.

## Conclusions

Preoperatively, a positive indication for systemic therapy is highly accurate for patients with early-stage breast cancer when compared to the postoperative (golden standard) indication. A negative preoperative indication is also highly accurate (96%), but only when exempting tumors larger than 2.0 cm on DCE-MRI or with PME>PDE ratios at ^31^P-MRS.
